# *Baylisascaris procyonis* Infection in Elderly Person, British Columbia, Canada

**DOI:** 10.3201/eid1802.111046

**Published:** 2012-02

**Authors:** Tawny Hung, Ronald C. Neafie, Ian R.A. Mackenzie

**Affiliations:** University of British Columbia, Vancouver, British Columbia, Canada (T. Hung, I.R.A. Mackenzie);; Armed Forces Institute of Pathology, Washington, DC, USA (R.C. Neafie)

**Keywords:** Baylisascaris procyonis, ascarid roundworm, parasites, Alzheimer disease, dementia, elderly person, Canada

**To the Editor:** *Baylisascaris procyonis* is an ascarid roundworm that commonly parasitizes the intestine of North American raccoons (*1**,**2*). As intermediate hosts, humans may develop visceral, ocular, and neural larva migrans (NLM) (*1**,**3*). Despite the high potential for exposure, only 20 cases of human cerebral *B. procyonis* infection have been reported, most causing devastating neurologic disease in young children (*1**,**4**–**6*). Here we expand the currently recognized spectrum of human disease by describing an unusual case of pathologically proven cerebral *B*. *procyonis* infection, which caused no apparent symptoms, in an elderly patient from British Columbia, Canada, with Alzheimer dementia.

The estimated prevalence of *B. procyonis* infection in North American raccoon populations is 68%–82% in the western United States (*2*) and 61% in southwestern British Columbia, Canada (*7*). Infected animals shed millions of *B. procyonis* eggs each day; the eggs embryonate in soil and are highly resilient in harsh environmental conditions (*1**,**2*). Raccoons tend to defecate in communal locations that are often in areas of human activity (*8*). Accidental or incidental human contact with contaminated soil may result in fecal-oral transmission. Following ingestion by an intermediate host, the infective larvae hatch in the small intestine and then penetrate the intestinal wall, migrate through the liver and lungs, and undergo extensive somatic dissemination (*1**–**3*). Migration causes mechanical tissue damage and provokes an inflammatory response. *B. procyonis* larvae grow in size but do not undergo further maturation in humans. The severity of disease is hypothesized to be related to the number of eggs ingested, the path of larval migration, and the extent of the host inflammatory response (*1**–**4*).

Despite the apparent potential for human disease, to our knowledge, only 20 cases of *B. procyonis* NLM have been reported (*1**,**4**–**6*). Most cases occurred in children <2 years of age or in older children and young adults with developmental delay. The only previously reported case of *B. procyonis* NLM in an adult was in a developmentally disabled 21-year-old adult known to exhibit geophagia and pica (*4*). Hemorrhagic necrotizing eosinophilic meningoencephalitis associated with large numbers of intact larvae has been described in patients with fatal cases (*1**,**4*), and all but 1 patient who survived (*9*) were left with severe neurologic deficits (*1**,**4**–**6*).

We report the case of a 73-year-old female nursing home resident with a 10-year history of moderately severe Alzheimer-type dementia. She was well-educated, had no other medical problems, and had previously resided with her husband in a rural part of British Columbia. Apart from mild confusion and poor memory, she was in good health and able to ambulate and communicate. There had been no recent change in her medical condition, and she voiced no concerns about medical problems before dying suddenly of cardiopulmonary arrest.

Autopsy findings showed a large pulmonary embolus as the cause of death. Mild, diffuse cerebral atrophy was the only gross brain abnormality (brain weight 1,210 g). Examination by microscopy was restricted to the brain and demonstrated Alzheimer-type pathology that was sufficiently severe to account for the patient’s dementia. In addition, sections of deep white matter from the left frontal lobe showed a small number of lesions, each consisting of a single larval nematode surrounded by mild chronic reactive changes and inflammation (macrophages, lymphocytes, plasma cells, and rare eosinophils) (Figure, panel A). Inflammation and reactive changes were restricted to the tissue immediately surrounding the larvae.

**Figure:**
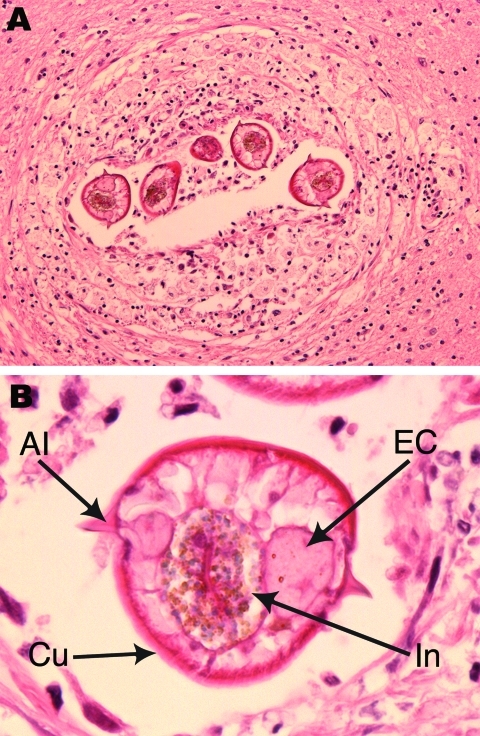
*Baylisascaris procyonis* infection in the frontal cerebral lobe white matter. A) Larval nematode seen in multiple transverse sections, surrounded by mild chronic inflammation and reactive changes. Hematoxylin and eosin stain; original magnification ×10. B) Morphologic features of the larvae included maximum diameter of 65 μm; thin, striated cuticle (Cu); single paired lateral alae (Al); and paired excretory columns (EC) that were smaller in diameter than the central intestine (In). Hematoxylin and eosin stain; original magnification ×40.

The larvae measured 65 μm in maximum transverse diameter and had a 1-μm thick striated cuticle that formed sharply pointed paired single lateral alae (Figure, panel B). The centrally located intestine measured 25 μm in diameter, was laterally compressed, and was lined by columnar cells with microvilli and abundant cytoplasmic granules. The intestine was flanked by smaller paired triangular excretory columns. There were 4–6 muscle cells per quadrant. These morphologic features are characteristic of *B. procyonis* roundworms and distinguish it from other nematodes that are known to affect humans (*2**,**3*).

This case is unusual in several respects: the patient is the oldest known person with confirmed *B. procyonis* NLM; it is only the second case reported from Canada (*5*); and it is a pathologically proven example of cerebral *B. procyonis* infection in a human without major clinical manifestations. Although this patient’s dementia could have masked subtle neurologic features, no changes were witnessed by caregivers or relatives, and the patient voiced no concerns. Her long-standing dementia was fully explained by Alzheimer-type pathology, and it is highly unlikely that the low-level and restricted anatomic distribution of parasitic infection would have contributed to her dementia. More likely, the combination of confusion and poor hygiene and ambulatory state in the patient may have predisposed her to acquiring *B. procyonis* roundworms through ingestion of contaminated soil. The existence of mild or subclinical *B. procyonis* brain infection is recognized in veterinary medicine (*1**,**2*). This case expands the currently recognized spectrum of human disease caused by *B. procyonis* roundworms to include mild or subclinical cerebral infection in elderly persons and suggests that dementia may increase the risk for exposure.

## References

[1] Gavin PJ, Kazacos KR, Shulman ST. Baylisascariasis. Clin Microbiol Rev. 2005;18:703–18. 10.1128/CMR.18.4.703-718.2005 16223954 PMC1265913

[2] Kazacos KR. *Baylisascaris procyonis* and related species. In: Samuel WM, Pybus MJ, Kocan AA, editors. Parasitic diseases of wild mammals. Ames (IA): Iowa State University Press; 2001. p. 301–41.

[3] Kazacos KR. Visceral, ocular, and neural larva migrans. In: Connor DH, Chandler FW, Schwartz DA, Manz HJ, Lack EE, editors. Pathology of infectious diseases. Vol. 2. Stanford (CT): Appleton and Lange; 1997. p. 1459–73.

[4] Murray WJ, Kazacos KR. Raccoon roundworm encephalitis. Clin Infect Dis. 2004;39:1484–92. 10.1086/425364 15546085

[5] Hajek J, Yau Y, Kertes P, Soman T, Laughlin S, Kanani R, A child with raccoon roundworm meningoencephalitis: a pathogen emerging in your own backyard? Can J Infect Dis Med Microbiol. 2009;20:e177–80. 21119798 10.1155/2009/304625 PMC2807255

[6] Perlman JE, Kazacos KR, Imperato GH, Desai RU, Schulman SK, Edwards J, *Baylisascaris procyonis* neural larva migrans in an infant in New York city. Journal of Neuroparasitology. 2010;1:1–5. 10.4303/jnp/N100502 PMC4205936 25346856

[7] Ching HL, Leighton BJ, Stephen C. Intestinal parasites of raccoons (*Procyon lotor*) from southwest British Columbia. Can J Vet Res. 2000;64:107–11. 10805249 PMC1189593

[8] Page LK, Anchor C, Luy E, Kron S, Larson G, Madsen L, Backyard raccoon latrines and risk for *Baylisascaris procyonis* transmission to humans. Emerg Infect Dis. 2009;15:1530–1. 10.3201/eid1509.090128 19788835 PMC2819851

[9] Pai PJ, Blackburn BG, Kazacos KR, Warrier R, Begue RE. Full recovery from *Baylisascaris procyonis* eosinophilic meningitis. Emerg Infect Dis. 2007;13:928–30. 17553240 10.3201/eid1306.061541

